# Epigenetics in liver disease

**DOI:** 10.1002/hep.27131

**Published:** 2014-05-07

**Authors:** Derek A Mann

**Affiliations:** Fibrosis Research Laboratories Institute of Cellular Medicine, Newcastle UniversityNewcastle upon Tyne, UK

## Abstract

Epigenetics is a term that encompasses a variety of regulatory processes that are able to crosstalk in order to influence gene expression and cell phenotype in response to environmental cues. A deep understanding of epigenetics offers the potential for fresh insights into the basis for complex chronic diseases and improved diagnostic and prognostic tools. Moreover, as epigenetic modifications are highly plastic and responsive to the environment, there is much excitement around the theme of epigenetic therapeutics, including not only new drugs but also more informed patient advice on lifestyle choices and their impact on pathology. This review briefly explains the molecular nature of the individual regulatory process that constitute epigenetics, including DNA methylation, histone modifications, chromatin remodeling, transcriptional control, and noncoding RNAs. The ways in which these epigenetic mechanisms influence liver physiology and disease will be considered in detail, particularly in the context of cancer, fibrosis, and nonalcoholic steatohepatitis. The current limitations associated with epigenetic profiling and therapeutics in liver disease are discussed, as is the intriguing possibility that environmental-induced epigenetic changes may become stable and heritable. *Conclusion*: The aim of the review is to inform hepatologists of the emerging key epigenetic ideas of relevance to liver diseases that are highly likely to form a component of patient management and care in the next decade. (Hepatology 2014;60:1418–1425)

An unanswered question in hepatology is why only a significant minority of liver patients progress to severe symptomatic states, whereas the majority remain relatively healthy. When we have the answers to this question patient management will be genuinely transformed, with anticipated advances in disease prognosis, patient stratification, and therapeutics. There are undoubtedly genetic influences which become ever more apparent from genome-wide association studies (GWAS). However, we still lack robust genetic explanations for population variability in the progression of liver disease to cirrhosis, hepatocellular carcinoma (HCC), and organ failure. Deeper GWAS may better illuminate the mechanistic basis for differential disease progression, as could the discovery of rare genetic polymorphisms that map to fibrogenesis and tumorigenesis. But additionally, there are many epigenetic influences on cell phenotype and disease. These signals operate literally above (“epi” meaning “upon” in Greek) the DNA sequence. Epigenetic mechanisms can operate at a genome-wide level to influence gene expression and cell behavior, they are highly dynamic, responsive to the cellular microenvironment, and exhibit considerable molecular diversity at the cellular, tissue, and organismal levels.

## Brief Description of Epigenetics and Its Constituent Regulatory Mechanisms

**Figure 1:**
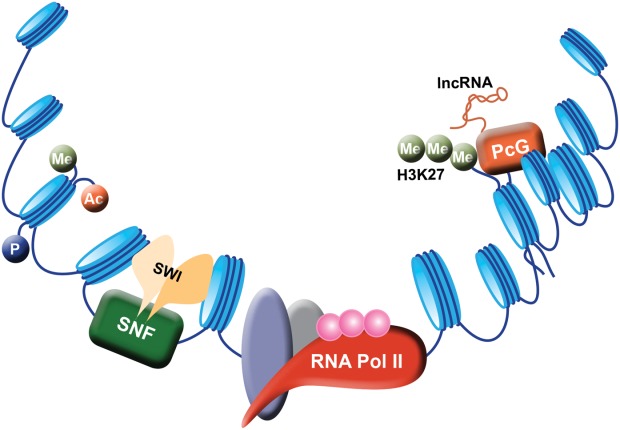
An overview of epigenetic mechanisms influencing gene expression. DNA is packed into histone octamers (or nucleosomes) that are depicted as “beads” or “spools” on the DNA “string.” The degree of compaction of nucleosomes at regulatory sequences is controlled by modifications to the histone tails such as phosphorylation (P), acetylation (Ac), and methylation (Me). Engagement of RNA polymerase II and associated transcriptional regulatory factors with chromatin at the gene promoter is reliant upon spacing and organization of nucleosomal structure as dictated by chromatin remodeling proteins (SWI/SNF). As transcription proceeds the ability of RNA polymerase II to read through the gene and elongate the nascent transcript is dependent on accessibility to downstream DNA, which is controlled by additional histone signatures. As an example, the presence of the signature H3K27me3 enables the recruitment of polycomb group complexes (PcG) which under the guidance of long noncoding RNAs (lncRNAs) can bring about chromatin compaction at specific loci. Such chromatin compaction in downstream regions of a gene will be inhibitory to RNA polymerase II transcriptional elongation leading to stalled or terminated transcription.

The consensus modern definition of an epigenetic trait is “*a stably inherited phenotype resulting from changes in a chromosome without alterations in the DNA sequence*.” By “inherited” this definition holds true for transmission of phenotype by both mitosis and meiosis. Most current reviews on epigenetics provide a narrow description usually focusing on three constituent regulatory systems; DNA (CpG) methylation, histone posttranslational modifications, and microRNAs (miRNAs). In fact, the reader should be aware of additional epigenetic influences such as transcription factors, histone remodeling complexes, and the entire gamut of noncoding RNAs, including the long noncoding RNAs (lncRNAs) that have recently emerged as regulators of chromatin structure and function (Fig. 1). Critically, there is substantial functional crosstalk between these distinct epigenetic elements, which combines to determine cell phenotype.

### DNA Methylation

CpG methylation is a common DNA modification that has a repressive influence on gene expression. It is regulated by DNA methyltransferases (DNMT1, DNMT3a, and DNMT3b) of which DNMT1 is a maintenance methyltransferase necessary for faithful copying of methyl-CpG marks to daughter DNA strands during mitosis.^1^ Methylated CpGs repress transcription by inhibiting the binding of transcription factors to DNA or by recruiting methyl-DNA binding proteins (MBDs) that influence chromatin structure.^1^ The recent discovery of the Tet enzymes that catalyze oxidation of methyl-CpG to generate hydroxymethyl-CpG has revealed the dynamic nature of DNA methylation.^2^ The hydroxymethyl-CpG modification is not only an intermediate step in the pathway to CpG demethylation, it also has its own regulatory properties and in contrast to methyl-CpG can stimulate gene transcription. DNA methylation has a major influence on phenotype; recent genome-scale DNA methylation profiling in three distinct human populations (Caucasian-, African-, Chinese-Americans) highlighted the contribution of differences in DNA methylation towards natural human variation.^3^

### Histone Code

DNA is packaged by histones into chromatin, which can take two forms: compacted transcriptionally inactive *heterochromatin* or lightly packaged transcriptionally permissive *euchromatin*. The basic structure of chromatin (Fig. 1) has been famously depicted as “beads on a string”; DNA being the *string* and the *beads* representing nucleosomes consisting of 147bp of double-stranded DNA (dsDNA) loosely wrapped around a core of eight histone molecules (two copies each of H2A, H2B, H3, and H4). The unstructured tail extensions of histones can be modified by phosphorylation at serine residues, methylation of arginine and by acetylation, methylation (mono, di, and tri), ubiquitination, sumoylation, and ADP-ribosylation at numerous lysine residues. Histone acetylation relaxes histone-DNA interactions and are associated with transcriptionally active chromatin. Histone lysine methylation plays a modulatory role in gene regulation and, depending on the lysine residue involved, will either suppress or promote transcription.^4^ Trimethylation of lysine 4 of histone 3 (H3K4me3) and H3K36me2/3 are usually associated with euchromatin. By contrast, H3K9me3 and H3K27me3 are associated with heterochromatin and silenced genes. However, the transcriptional activity of a gene is determined by the cumulative influences of multiple histone modifications (or a histone “code”). The histone code is actively involved in control of cell phenotype, is highly dynamic, and under the regulatory control of enzymes that either add (“writers”) or remove (“erasers”) posttranslational modifications. The code is then interpreted by mediator proteins (“readers”) that affect histone-DNA interactions and nucleosomal organization.^4^ Nucleosome structure can also be regulated by the exchange of core histones with one or more histone variants.^5^ As an example, exchange of H2A for H2A.Z is functionally important in gene activation and silencing.

### Chromatin Remodeling and Compaction

The dense spacing and packaging of nucleosomes needs to be remodeled to allow access to transcription factors. This is carried out by the activities of adenosine triphosphate (ATP)-dependent chromatin remodeling complexes (e.g., mammalian SWI/SNF or BAF) that alter nucleosome-DNA contacts, promote nucleosome repositioning, or regulate the incorporation of variant histones into the nucleosome.^6^ By contrast, silencing of genes involves compaction of nucleosomes into dense chromatin. The polycomb group (PcG) proteins represent a global gene silencing system that plays a critical role in cell determination and fate. PcG proteins contribute to two multiprotein complexes known as polycomb repressor complexes 1 and 2 (PRC1 and PRC2). PRC2 and its constituent enzyme EZH2 stimulate H3K27 trimethylation^7^; this epigenetic mark recruits PRC1, a stimulator of chromatin compaction. Loss of PcG function is implicated in cancers, in particular, EZH2 is overexpressed in many human cancers where it silences expression of tumor suppressor genes such as the Ink4/Arf locus.^7^

### Transcription Factors

Some transcription factors can exert a global influence on gene expression and cell phenotype in the liver, including members of the PPAR and CEBP transcription factor families and regulators of the circadian clock (e.g., CLOCK/BMAL) involved in control of hepatic metabolism and bile synthesis. These proteins are important in the epigenetic machinery since they are directly wired into signaling events that are downstream of extracellular receptors for environmental cues such as microbes, nutrients, hormones, growth factors, and xenobiotics. Moreover, a great many transcription factors engage in crosstalk with the chromatin regulatory machinery through their direct interaction with coactivators (e.g., histone acetyltransferases) or corepressors (e.g., histone deacetylases) at target genes. Of relevance to hepatologists, the bile acid sensor farnesoid X receptor (FXR) regulates gene transcription in cooperation with a number of coregulators such as SRC-1, SIRT1, Brg-1, CARM1, PRMT1, and N-coR.^8^

### Noncoding RNAs (ncRNAs)

Until recently, the majority of the noncoding genome was thought to be “junk” DNA, but we are now aware that it carries important regulatory information transmitted by way of the ncRNAs. Most readers will be familiar with miRNAs, which are short (22 nucleotide) molecules that function in gene silencing and have already been manipulated for the design of antivirals or as cancer drug targets.^9^,^10^ It is important to also be aware of the long-noncoding RNAs (lncRNAs), which are anticipated to be extremely abundant and that are generated from a complex network of overlapping sense and antisense transcripts often including protein-coding loci.^11^,^12^ The lncRNAs are implicated in almost every step of gene regulation including transcription, splicing, and translation. Furthermore, lncRNAs are able to recruit chromatin-modifying complexes to specific genomic loci, thus playing a fundamental epigenetic role. Not unexpectedly, relationships between lncRNAs and a variety of human diseases including HCC are emerging.^12^

## Epigenetics and Hepatocellular Carcinoma

### DNA Methylation and HCC

Typical epigenetic lesions in liver cancer include genome-scale changes in the DNA methylation landscape, loci-specific DNA hypermethylation, dysfunction of histone-modifying enzymes, and abnormal expression of ncRNAs. Cancer-related changes in DNA methylation are attractive as biomarkers since they can be readily detected and quantified from fixed tissues. As a consequence, there are many published studies reporting DNA methylation patterns specific to liver cancers of different etiologies, including recent genome-wide studies. Combined DNA methylation and transcriptome mapping in human HCC identified 230 genes whose promoters were hypomethylated and had elevated expression in HCC (epigenetically induced), and 322 genes that were hypermethylated and underexpressed in tumors (epigenetically repressed).^13^ Epigenetically induced genes were mapped to pathways driving cellular differentiation and transformation, tumor growth, and metastasis. Repressed genes mapped to apoptosis, cell adhesion, and cell cycle progression. A study of hepatitis B virus (HBV)-induced HCC compared DNA methylation profiles between tumor and adjacent tissue, this identified 1,640 hypomethylated and 684 hypermethylated CpG in the tumor.^14^ Using a similar approach, Song et al.^15^ reported that 62,692 loci displayed differential methylation between HCC and surrounding tissue, of which a remarkable 61,058 were hypomethylated. In a more focused study, tumor suppressor genes were identified that are hypermethylated in the early stages of HCC.^16^ Eight genes (*HIC1, GSTP1, SOCS1, RASSF1, CDKN2A, APC, RUNX3* and *PRDM2*) displayed significantly increased methylation in early HCCs and were associated with shorter-time-to-HCC occurrence.

Despite the excitement surrounding genome-wide DNA methylation studies, there are a number of caveats to be considered that urge caution regarding the clinical and biological significance of the emerging datasets. Perhaps of most importance is that tumors have high cellular heterogeneity, as such observed differences in DNA methylation patterns may simply reflect differences in numbers of tumor to normal cells rather than identifying epigenetic signatures relevant to cancer biology. For DNA methylation profiling to deliver definitively relevant clinical data, it will be necessary to carry out quantitative analysis on small numbers of histologically verified tumor cells captured from tissue by a technique such as laser dissection microscopy or high-speed cell sorting. A further caveat is that we must not assume a simple relationship between changes in DNA methylation and altered gene expression, even where this is indicated by overlaid transcriptome data. A direct functional correlation would require *in vivo* experimental manipulation of DNA methylation in a site-directed manner and demonstration of an associated change in the rate of gene transcription.

### Viruses as Drivers of Epigenetic Changes Underlying HCC

Cancers of viral origin can provide insights into relationships between epigenetics and tumor biology. The oncogenic HBx protein of HBV induces the expression of DNMT1 and recruits DNMT1, 3a, and 3b to stimulate hypermethylation of *IGFBP-3* and *p16^INK^*.^17^ One mechanism by which HBx has been proposed to induce DNMT1 is by down-regulating microRNA miR-152, which directly targets the DNMT1 transcript.^18^ Overexpression of miR-152 results in global DNA hypomethylation, whereas inhibition of miR-152 caused global hypermethylation and increased DNA methylation at the *GSTP1* and *CDH1* tumor suppressor genes. HCV has also been shown to stimulate alterations in DNA methylation; for example, the Gadd45β promoter is hypermethylated in HCV transgenic mouse liver and in cells infected with the JFH1 strain of HCV.^19^ Gadd45β is expressed at reduced levels in HCV-infected patients and in tumor tissue; this is functionally significant given the role of Gadd45β in the control of cell cycle, growth arrest, and DNA repair. Studies in HBV- and HCV-induced HCC have identified common functional mutations in the SWI/SNF-like ATP-dependent chromatin remodeling enzymes *ARID1A* and *ARID2*.^20^–^22^ Exome sequencing in an HCC tumor and adjacent nontumor tissue of HBV/HCV origin discovered missense mutations in genes encoding the H3K4 methyltransferases *MLL, MLL2, MLL3*, and *MLL4*.^23^ These enzymes are important for remodeling chromatin into a transcriptionally active state. *MLL4* is of particular interest, being a recurrent hotspot for HBV integration and considering its role as a regulator of p53 target genes.^23^

**Figure 2:**
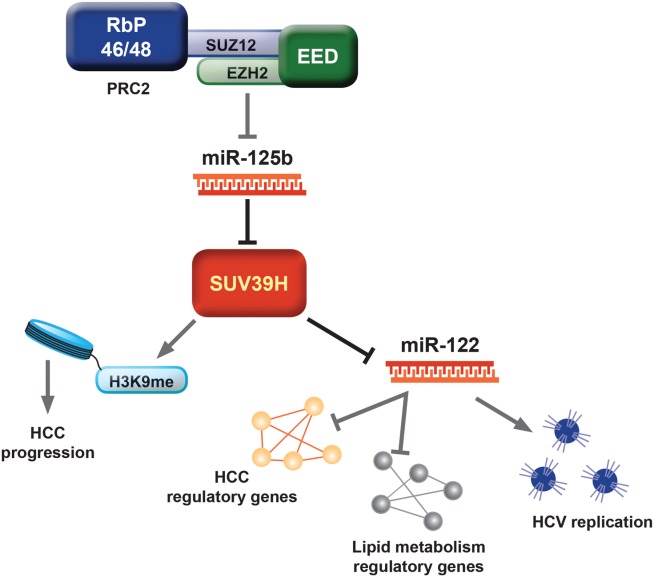
An example of complex epigenetic crosstalk and its impact on liver physiology and disease. The histone methyltransferase SUV39H plays a central regulatory role in liver physiology; on the one hand, negatively regulating the expression of the microRNA miR-122, which orchestrates the epigenetic control of gene networks involved in lipid metabolism and HCC. In addition, miR-122 is critical in the life cycle of HCV and is considered a therapeutic target. On the other hand, SUV39H regulates histone modifications at genes encoding regulators of cell proliferation and migration, its overexpression is associated with HCC. Expression of SUV39H is posttranscriptionally regulated by miR-125, which in turn is transcriptionally under the influence of the polycomb group complex PRC2 and its H3K27 methyltransferase EZH2.

The PRC2 methyltransferase EZH2 and its structural partners EED, SUZ12, and RBP7 are expressed at elevated levels in human HCC and contribute to tumorigenesis by silencing multiple miRNAs.^24^ The PRC2-regulated miR-125b is a transcriptional corepressor of the H3K9 methyltransferase SUV39H (Fig. 2), which regulates heterochromatin formation.^25^ SUV39H is overexpressed in human HCC and when knocked-down in HCC cell lines inhibits proliferation and migration. SUV39H is a repressor of miR-122, which is best known for its ability to stimulate translation of HCV RNA (Fig. 2). However, miR-122 is decreased in HBV infection and when genetically deleted in mice results in spontaneous hepatosteatosis, inflammation, fibrosis, and HCC.^26^,^27^ HBx represses miR-122 by recruiting peroxisome proliferator-activated receptor gamma (PPARγ) and its associated SUV39H-containing corepressor complex to the miR-122 promoter.^28^ Hence, altered expression or mutation of histone methyltransferase genes in HCC disrupts multiple regulatory networks, including a large number of miRNAs involved in posttranscriptional control. Drugs that modulate the activity of one or more of these epigenetic enzymes may be of considerable therapeutic potential.

A number of miRNAs are of mechanistic relevance in HCC and are described in detail elsewhere.^14^ LncRNAs have so far received considerably less attention; however, several are emerging as potentially important in HCC. Highly Up-regulated in Liver Cancer (HULC) is a 500 nucleotide lncRNA which was discovered from a screen of noncoding RNAs expressed in HCC. HULC is expressed in normal human hepatocytes but is strongly induced in HCC tissue.^29^ Elevated HULC expression is also a feature of HBV infection^30^ and is found in liver metastatic tissue of colorectal cancer origin. HULC regulates HCC proliferation and expression of a number of HCC-associated genes and is detected in the sera of HCC patients, the latter raising potential for biomarker development. HOTAIR is expressed in HCC and is associated with a higher risk of tumor recurrence following therapeutic transplantation.^31^ Depletion of HOTAIR inhibits tumor cell proliferation, stimulates apoptosis, and generates significant antitumor effects *in vivo*.^32^ MALAT1 is a very large (8,000 nt) nuclear lncRNA expressed in HCC and is associated with high risk of posttransplant recurrence.^33^ Knockdown of MALAT1 in HCC cell lines has similar behavioral effects to those described for depletion of HOTAIR. Future work with lncRNAs is greatly anticipated and expected to lead to exciting new insights into hepatic gene regulation.

## Epigenetics and Liver Fibrosis

Hepatic stellate cell (HSC) transdifferentiation to a profibrogenic myofibroblastic phenotype is a pivotal event in fibrogenesis. Transdifferentiation requires global epigenetic remodeling to bring about the suppression of adipogenic differentiation factors, *de novo* expression of regulators of the myofibroblast phenotype, and cell cycle entry. According to Waddingtons famous epigenetic landscape model,^34^ reversion or conversion of a differentiated state is energetically costly for the cell and this ensures the stability of cell phenotype and tissue organization. Hence, the HSC may need to overcome energy-dependent epigenetic barriers to adopt the myofibroblast phenotype. In this respect, it is noteworthy that autophagy, a mechanism by which the cell recycles its intracellular components to generate energy, is critical for HSC activation.^35^ Small molecular epigenetic inhibitors such as the DNMT1 inhibitor 5-azadeoxycytidine (5-AzadC) and the EZH2 inhibitor 3-deazaneplanocin A (dZNep) potently inhibit HSC activation *in vitro* and *in vivo*.^36^,^37^ Studies in our laboratory have described an epigenetic relay pathway that must be activated in order to drive HSC transdifferentiation.^37^ Mice lacking MeCP2 are protected from liver fibrosis and *mecp2-*deficient HSC display multiple defects in their fibrogenic phenotype including reduced expression of collagen I, TIMP-1, and α-SMA. MeCP2 may be a generic core-regulator of tissue fibrosis since *mecp2*-deleted mice are also protected from pulmonary fibrosis.^38^ MeCP2 operates two concurrent mechanisms to ensure epigenetic silencing of PPARγ and HSC transdifferentiation. MeCP2 directly binds to methyl-CpG-rich regulatory regions in the PPARγ promoter and recruits H3K9me3-modifying enzymes that suppress transcription initiation. MeCP2 is also required for expression of EZH2 and H3K27me3 modifications in the downstream coding region of the gene that impede transcriptional elongation. These two mechanisms help explain the ability of 5AzadC and dZNep to inhibit HSC transdifferentiation. More recently, our laboratory described how MeCP2 can promote transcription of multiple profibrogenic genes through its control of the expression of the H3K4/H3K36 methyltransferase ASH1.^39^

Changes in DNA methylation during HSC activation have been reported at specific loci such as the PTEN tumor suppressor and Patched 1 (PTCH1) genes; in both cases the genes become hypermethylated and this corresponds with diminished expression in the myofibroblast.^40^ To date, we lack genome-wide studies of changes in DNA methylation during HSC activation. However, a landmark study recently published from the Diehl laboratory interrogated 69,247 differentially methylated CpG sites in liver biopsy material from nonalcoholic fatty liver disease (NAFLD) patients stratified into advanced (F3-4) versus mild (F0-1) disease.^41^ 76% of the differentially modified CpG sites became hypomethylated in advanced disease, while 24% underwent hypermethylation. The mechanistic basis for these NAFLD-associated changes in DNA methylation was not investigated. The DNA methylome data were overlaid with transcriptomics data from the same biopsies; this led to the discovery of several key fibrogenic genes that were both hypomethylated and overexpressed in advanced NAFLD. However, when using whole tissue for DNA methylome analysis there is a risk that observed differences may simply be reflecting cellular and/or architectural changes in the tissue rather than identifying molecular changes that are driving fibrogenesis. There is also no proof that the alterations in DNA methylation are directly responsible for the observed differences in gene expression; the two may simply be coincidental.

It is highly likely that noncoding RNAs of all sizes and activities will play fundamental functions in the determination of HSC phenotype and liver fibrosis. Numerous miRNAs regulating proliferation, apoptosis, TGFβ1 signaling, and collagen expression have been described as regulators of HSC phenotype and fibrosis progression.^42^ We await investigations into the functions of lncRNA species.

## Epigenetics and Nonalcoholic Steatohepatitis (NASH)

The associations between nutrition, epigenetics, and metabolic disease are firmly established. The phenotype of the Agouti mouse, which includes obesity and predisposition to cancer, is prevented by supplementation of the maternal diet with methyl donors.^43^ Diets depleted of methyl donors promote DNA hypomethylation and the development of steatosis in rodents. By contrast, supplementation of high-calorie diets with methyl donors prevents NAFLD, suggesting that epigenetic changes that alter hepatic fat metabolism may be related to dynamic alterations in DNA methylation.^44^,^45^ There is a close association between lipid metabolism and circadian rhythm, the latter being controlled by the CLOCK machinery. The CLOCK-BMAL1 circadian transcription factors regulate hundreds of genes including the PPARs; hence, metabolic genes regulated by PPARs are rhythmically expressed.^46^ Mice lacking the expression of *Clock* are hyperphagic, obese, and develop NASH.^47^ The deacetylase SIRT1 forms a chromatin complex with CLOCK-BMAL1 and its activity is regulated in a circadian manner. This CLOCK-SIRT complex determines the degree of histone acetylation and amplitude of transcription for circadian and metabolic genes. Moderate overexpression of *Sirt1* in mice protects from high-fat-diet-induced metabolic disease.^48^ These data are of relevance when considering epidemiological data in humans with disturbed circadian rhythms such as shift workers who have a high risk of metabolic disorders.^49^ However, clinical studies investigating epigenetic reprogramming in NASH are only just beginning to emerge. Ahrens et al.^50^ carried out DNA methylation and transcriptome analysis on liver biopsies from lean controls, healthy obese, and NASH patients. Analysis of 45,000 CpG sites revealed 467 dinucleotides where methylation deviated from lean controls. By overlay with transcriptome data, eight genes were identified (*GALNTL4, ACLY, IGFBP2, PLCG1, PRKCE, IGF1, IP6K3* and *PC*) that displayed obesity-related alterations in expression correlating in an inverse manner with altered CpG methylation. Noteworthy is that all of these genes are regulators of metabolism and candidates for NAFLD disease drivers. As part of the same study, a similar analysis was also carried out with paired liver biopsies obtained 5-9 months following bariatric surgery. The gene encoding protein-tyrosine phosphatase epsilon (*PTPRE*), a negative regulator of insulin signaling, became hypermethylated and transcriptionally down-regulated with weight loss. This finding provides an interesting mechanistic link between weight loss and control of hepatic insulin sensitivity.

Progression to steatohepatitis is a critical step in the continuum of NAFLD towards fibrotic disease and/or HCC. A plausible hypothesis for progression of benign steatosis to steatohepatitis is the perturbed regulation of inflammatory cytokines (e.g., interleukin [IL]-6, IL-1, and tumor necrosis factor alpha [TNFα]). Hepatocytes cultured with free fatty acids overexpress the ATP-dependent chromatin remodeling proteins Brg1 and Brm, which upon recruitment to proinflammatory genes stabilize nuclear factor kappa B (NF-κB) binding and help remodel chromatin.^51^ Impressively, experimental depletion of Brg1 into MCD-fed mice suppressed steatosis, inflammation, and fibrosis, indicating a pivotal role for Brg/Brm chromatin remodeling proteins in the progression of NAFLD.

Investigations of noncoding RNAs in NASH have so far been limited to miRNAs. On the order of 100 miRNAs are reported to be differentially expressed in NASH and these have vast functional diversity, including control of lipid and glucose metabolism.^52^ Noteworthy is miR-122, which plays important regulatory functions in lipid and cholesterol metabolism and is closely linked to the circadian clock system. mIR-122 is abundantly expressed in healthy liver but down-regulated in NASH, and in experimental studies in mice has been functionally implicated in NAFLD pathology.^53^

## Epigenetics and Liver Disease Imprinting

The concept that environmental cues may induce stable adaptive traits that can be passed between generations and influence phenotype has its theoretical roots in so-called “Lamarckian” inheritance. Until recently, there was limited convincing experimental evidence for epigenetic transgenerational effects in mammals and most studies were of maternal origin, where it can be argued that contributory *in utero* events confound data interpretation. By contrast, paternal effects are more attributable to true epigenetic inheritance, as fathers usually only contribute sperm to offspring. Of relevance, there are intriguing recent reports for paternally transmitted heritable epigenetic adaptations that impact on liver function. Male inbred mice fed a low-protein diet gave rise to offspring that exhibited elevated hepatic expression of genes regulating lipid and cholesterol metabolism.^54^ Liver fibrosis in male outbred rats triggers the multigenerational transmission of changes in the expression of genes regulating hepatic stellate cell activation, with the phenotypic consequence being suppression of liver fibrosis.^55^ Interestingly, in both of these studies DNA methylation and gene expression for PPARα and PPARγ were altered; this may indicate that these nuclear hormone receptors provide an epigenetic hub for integrating ancestral environmental information. Evidence for Lamarckian-like inheritance is rare in humans, although intergenerational inheritance of metabolic disorders following the 1944-1945 Dutch famine does provide a striking example. As reported by Veenendaal et al.,^56^ body mass index (BMI) and weight are increased in F2 (grandchildren) of males exposed to *in utero* famine, suggesting that epigenetic adaptations to dietary factors may be stable and transmissible across multiple generations. Whether such ancestral epigenetic imprinting contributes to population variability in liver diseases remains to be determined.

## Summary and Future Prospects

Genetic and epigenetic variants combine to influence observed differences in disease susceptibility and variable disease progression. Based on the experience with GWAS, it is inevitable that epigenome-wide association studies (EWAS) in liver diseases will be undertaken. However, conducting meaningful EWAS will be challenging, as epigenetic signatures are highly plastic, display differences between cells within a tissue, and are modified by aging and multiple environmental factors. But if carefully conducted, EWAS combined with GWAS offers the rewards of unparalleled mechanistic insights into disease pathology, improved patient stratification, and new prognostic tools. Unlike genetic drivers of disease, unhealthy epigenetic modifications may be modified, thus offering the prospect of epigenetic therapies. There are already ongoing clinical trials with HDAC inhibitors in cancer, the miR-122 inhibitor miravirsen in chronic HCV, and first-into-man trials with a mimetic of miR-34, a powerful tumor suppressor.^57^,^58^ But again, enthusiasm must be tempered with what are significant hurdles to be overcome, not the least in finding drugs with sufficient specificity for a given epigenetic modifier to ensure efficacy and prevent clinical toxicities. Notably, very few HDAC inhibitors have completed phase II testing due to adverse side effects including fatigue, constipation, diarrhea, and dehydration.^59^ Ideally, combined GWAS and EWAS information will arm clinicians and dieticians with the tools to design evidence-based lifestyle modification strategies tailored to prevent liver disease developing in at-risk individuals and from passing on unhealthy epigenetic traits to future generations.

## Abbreviations

GWAS: genome-wide association studies
lncRNAs: long noncoding RNAs
MBD: methyl-DNA binding protein
miRNAs: microRNAs
PcG: polycomb group
